# Low-Density Polyethylene-Based Novel Active Packaging Film for Food Shelf-Life Extension via Thyme-Oil Control Release from SBA-15 Nanocarrier

**DOI:** 10.3390/nano14050423

**Published:** 2024-02-26

**Authors:** Aris E. Giannakas, Maria Baikousi, Vassilios K. Karabagias, Ioanna Karageorgou, George Iordanidis, Charmpas Emmanouil-Konstantinos, Areti Leontiou, Andreas Karydis-Messinis, Nikolaos E. Zafeiropoulos, George Kehayias, Charalampos Proestos, Constantinos E. Salmas

**Affiliations:** 1 Department of Food Science and Technology, University of Patras, 30100 Agrinio, Greece; vkarampagias@upatras.gr (V.K.K.); i.karageorgou@upatras.gr (I.K.); up1076521@ac.upatras.gr (G.I.); aleontiu@upatras.gr (A.L.); gkechagi@upatras.gr (G.K.); 2 Department of Material Science and Engineering, University of Ioannina, 45110 Ioannina, Greece; mbaikou@uoi.gr (M.B.); emmanouil.charmpas@gmail.com (C.E.-K.); a.karyd@uoi.gr (A.K.-M.); nzafirop@uoi.gr (N.E.Z.); 3 Laboratory of Food Chemistry, Department of Chemistry, National and Kapodistrian University of Athens Zografou, 15771 Athens, Greece; harpro@chem.uoa.gr

**Keywords:** SBA 15, nanocarrier, thyme oil, LDPE, active packaging, food preservation, control release, natural preservatives, antioxidant activity, pork meat shelf-life extension

## Abstract

The use of natural raw substances for food preservation could provide a great contribution to food waste reduction, circular economy enhancement, and green process application widening. Recent studies indicated that the use of porous materials as adsorbents for natural essential oils provided nanohybrids with excellent antioxidant and antimicrobial properties. Following this trend in this work, a thymol oil (TEO) rich SBA-15 nanohybrid was prepared and characterized physiochemically with various techniques. This TEO@SBA-15 nanohybrid, along with the pure SBA-15, was extruded with low-density polyethylene (LDPE) to develop novel active packaging films. Results indicated that TEO loading was higher than other porous materials reported recently, and the addition of both pure SBA-15 and TEO@SBA-15 to the LDPE increased the water/oxygen barrier. The film with the higher thyme-oil@SBA-15 nanohybrid content exhibited a slower release kinetic. The antioxidant activity of the final films ignited after 48 h, was in the range of 60–70%, and was almost constant for 7 days. Finally, all tests indicated a sufficient improvement by the addition of thyme-oil@SBA-15 nanohybrids in the pure LDPE matrix and the concentration of wt. 10% of such nanocarriers provided the optimum final LDPE/10TEO@SBE-15 active packaging film. This material could be a potential future product for active packaging applications.

## 1. Introduction

The substitution of food chemical preservatives with antioxidant and antimicrobial substances abundant in nature is of major interest nowadays. The decrease in food waste, the positive environmental fingerprint, the circular economy, and sustainability are parameters that affect the industrial food packaging sector. Such parameters indicated the use of natural bioactive agents as a remarkable tool for the transition from classic to novel active food packaging methods [1,2,3,4,5]. This means that, in the future, the foods should not only be protected but also preserved for a longer period before being consumed or wasted. The most trendy and promising method for this achievement is to use bio-based materials [3,5,6,7]. Such food additives are naturally abundant antioxidant/antibacterial phytochemical extracts and essential oils (EOs) [8,9]. Recent research has indicated that the inclusion of such natural extracts and EOs as antioxidant/antibacterial compounds in porous solids and the controlled release of them in the food [10,11] resulted in better food preservation conditions. Such natural extracts and EOs are generally recognized as safe (GRAS), which makes consumers and regulatory agencies consider them more appropriate for use in food than artificial preservative compounds such as butylated hydroxyanisole (BHA) and butylated hydroxytoluene (BHT) [12]. In the last few years, essential oils (EOs) and their components have been the most widely used, naturally abundant antioxidant and antibacterial compounds for active food packaging applications.

Chemical components of the thyme essential oil (TEO) include monoterpenes, monoterpene alcohols, phenol derivatives, ketones, aldehydes, ethers, and esters [13]. Recently, many studies have concluded that, during the shelf-life period, the use of TEO increases stability and reduces lipid oxidation of various food products such as meat, meat products, milk, fish, or fish products. This observation indicates that TEO is a promising source of natural additives. The main components of TEO are the isomeric phenolic monoterpenes thymol (2-isopropyl-5-methylphenol) and carvacrol (2-methyl-5-(propan-2-yl)phenol) [13]. Many polymers and biopolymers-based active films have been developed, characterized, and applied as active food packaging films [14,15,16]. Among the various polymers and biopolymers used for such active packaging films, low-density polyethylene (LDPE) has been extensively used due to its flexibility and good water barrier properties [16,17]. 

To avoid direct loss of EOs, from the active package due to their volatile nature nanocarriers such as montmorillonite nanoclay, halloysite tubular nanoclay, natural zeolite, and activated carbon have been suggested to adsorb them and control their release from the package to food [18,19,20,21,22,23,24]. In this direction, mesoporous silica could also be a promising nanocarrier for such EOs because of its large pore size and its high specific surface area [25,26,27]. Recently, it has been shown that the release of drugs or other pre-adsorbed molecules from mesostructured silicas depends on the pore architecture, the pore size, and the specific drug-silica pore wall interactions [28,29]. Among the various mesoporous silicas, SBA (Santa Barbara Amorphous)-15 is characterized by hexagonally packed one-dimensional nanochannels and is one of the most studied in terms of drug delivery properties [29,30,31,32,33]. Garguilo et al. [30] adsorbed alpha-tocopherol in SBA-15 and prepared LDPE-based antioxidant films. Experimental measurements have shown that alpha-tocopherol release from polymer films was slower, and the alpha-tocopherol antioxidant effectiveness was higher when adsorbed on modified SBA-15. Gamez et al. [26] loaded thymol into SBA-15 nanoparticles and incorporated the produced nanohybrids in polycaprolactone (PCL) electrospun nanofibers. Control release studies have shown that the obtained PCL-based fibers contained 5.6 wt.% of thymol, and more than half of this loading was released in the first 7 h. This release prevented initial bacterial colonization and inhibited or eliminated bacterial growth, as shown in carrying out in vitro experiments against *Staphylococcus aureus* [26].

In our current study, TEO was adsorbed in SBA-15 mesoporous silica to develop a novel, nanohybrid, TEO@SBA-15 which was then incorporated into low-density polyethylene (LDPE) matrix via a melt extrusion process to obtain LDPE/xTEO@SBA-15 active packaging films for the first time (where x = 5, 10, and 15 and means 5, 10, and 15 wt.% of TEO@SBA-15 addition in LDPE). Furthermore, LDPE/xSBA-15 (where x = 5, 10, and 15) were also developed for comparison. Such LDPE/xSBA-15 and LDPE/xTEO@SBA-15 films are reported for the first time. The TEO@SBA-15 nanohybrid was physiochemically characterized with X-ray diffraction (XRD) measurements, Fourier Transform Infrared (FTIR) spectroscopy, Differential Scanning Calorimetry (DSC), Thermogravimetric analysis (TGA) experiments, and TEO control release kinetics. The obtained LDPE/xSBA-15 and LDPE/xTEO@SBA-15 films were physiochemically characterized with XRD, FTIR, tensile properties, and Dynamic Mechanical Analysis (DMA) measurements. Barrier capabilities of such novel LDPE/xSBA-15 and LDPE/xTEO@SBA-15 packaging films against water and oxygen transport through films were also evaluated via calculations of the water vapor diffusion (D_wv_) and oxygen permeability (P_O2_) coefficients using Water Vapor Transmission and Oxygen Transmission Rate (WVTR/OTR) experimental measurements. The antioxidant potential of future food packaging was estimated by calculating the total antioxidant activity of these films as well as the 50% effective concentration (EC50) values of the antioxidant activity of the films. TEO control release kinetics, such as the kinetic constant (k) of TEO release from the films as well as the diffusion coefficient of TEO (D_TEO_) inside the film, were calculated utilizing the gravimetrically measured wt.% values for the total TEO release amount from the films. Finally, fresh pork fillets were wrapped with pure LDPE, the optimum LDPE/10TEO@SBA-15 films were obtained, and a shelf-life experiment was carried out. The lipid oxidation values were estimated via the Thiobarbituric Acid Reactive Substances method (TBARS), the heme iron content values, the Total Variable Counts (TVCs), and the sensory values. 

## 2. Materials and Methods

### 2.1. Materials

Chemco company (Via Achille Grandi, 13—13/A, 42030 Vezzano sul Crostolo RE, Italy) was the supplier of the Thyme Essential Oil (TEO). Sigma-Aldrich (Sigma-Aldrich, St. Louis, MO, USA) was the supplier of Tetraethyl orthosilicate (TEOS) 98% with CAS no. of 78-10-4, triblock copolymer Pluronic P123 (EO20PO70EO20, EO = ethylene oxide, PO = propylene oxide) with CAS No. of 9003-11-6, Hydrochloric acid 37% with CAS No. of 7647-01-0, LDPE with a CAS no. of 9002-88-4 and extra pure DiPhenyl-1-PicrylHydrazyl (DPPH) with CAS no. of 1898-66-4. Merck Company (KgaA 64271 Darmstadt, Germany) was the supplier of the pro-analyze Thio Barbituric Acid (TBA), while Fisher Scientific Company (Bishop Meadow Road, Loughborough, LE11 SRG, UK) was the supplier of Acetone 99%. Three samples of “skalopini”-type pork meat with no bones and weighing 700 g each were donated by the local Greek company Aifantis (Aifantis Group, Acheloos Bridge, Agrinio, Greece 30100) one hour after slaughter.

### 2.2. Preparation of SBA-15

The SBA-15 was synthesized following the recipe provided by Mavrogiorgou et al. [34]. Triblock copolymer surfactant pluronic P123 was incorporated with Tetra Ethyl Ortho Silicate (TEOS), which was used as a silica source. In more detail, an amount of 4.0 g of P123 copolymer was diluted into 150 g of 1.6 M HCl. Sequentially, 8.50 g of TEOS was incorporated into the prepared solution, and the mixture was stirred until TEOS was dissolved. The final solution was heated under static conditions, first at 311 K for 22 h and subsequently at 368 K for 24 h. The product was filtered without washing and dried in the air. The template was removed by calcination at 773 K for 6 h with a rising heating rate of 1.5 K/min.

### 2.3. Preparation of TEO@SBA-15 Nanohybrid

Three grams of as-prepared SBA-15 were dried at 120 °C under vacuum. Then, in the dried SBA-15, approximately 10 mL of TEO was added drop by drop and stirred until a slurry was obtained. The obtained slurry was then stirred overnight since the excess TEO evaporated. The obtained TEO@SBA-15 nanohybrid powder was stored for further use.

### 2.4. Preparation of LDPE/xSBA-15 and LDPE/xTEO@SBA-15 Films

**Figure 1:**
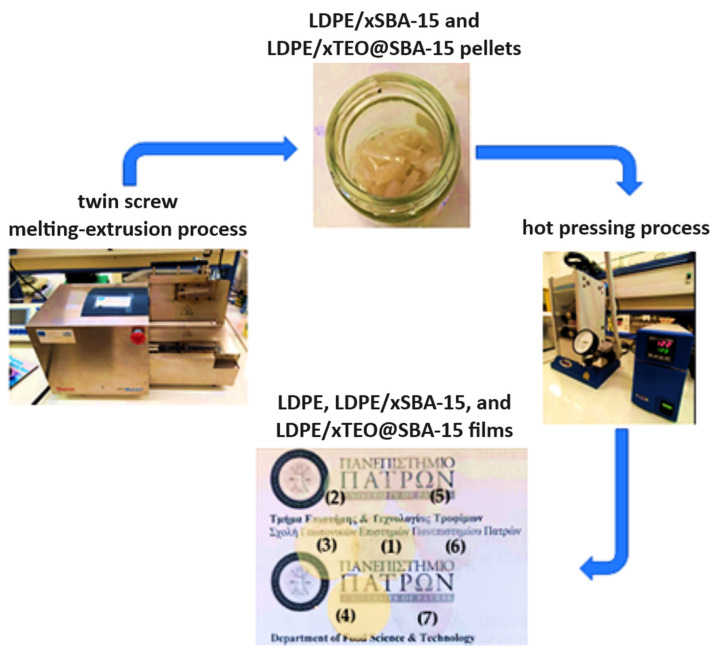
A schematic presentation of the process followed for the preparation of (1) LDPE, (2) LDPE/5SBA-15, (3) LDPE/10SBA-15, (4) LDPE/5SBA-15, (5) LDPE/5TEO@SBA-15, (6) LDPE/10TEO@SBA-15, and (7) LDPE/15TEO@SBA-15 films.

A Mini Lab twin-screw extruder (Haake Mini Lab II, Thermo Scientific, ANTISEL, S.A., Athens, Greece) was operated at 140 °C and 100 rpm screw rotation for 3 min to produce LDPE/xSBA-15 and LDPE/xTEO@SBA-15 films [24]. Appropriate amounts of LDPE granules, SBA-15 powder, and TEO@SBA-15 powder were mixed to achieve final LDPE/xSBA-15 and LDPE/xTEO@SBA-15 materials where x = 5, 10, and 15 wt.% nominal concentrations. A pure LDPE sample was also prepared using the twin-screw extruder. The materials obtained after the extrusion process were transformed into films via a thermomechanical process using a hydraulic press with heated platens. Approximately 1.4 g of pellets were heated/pressed at 110 °C under a constant pressure of 2 MPa to obtain films with a 10 cm diameter and an average thickness of 0.10–0.25 mm. The overall process is depicted in Figure 1.

### 2.5. Physicochemical Characterization of SBA-15, and TEO@SBA-15 Nanohybrid

The methods used for the physicochemical characterization of SBA-15 as received and, TEO@SBA-15 obtained nanohybrid are described in detail in previous recent publications [21,23,24]. Briefly, to study possible crystallinity changes in the SBA-15 during the modification process to obtain the TEO@SBA-15 nanohybrid, the XRD instrumental analysis technique was employed. Both pure SBA-15 and TEO@SBA-15 powders were analyzed using a Bruker D8 Advance diffractometer (Brüker, Analytical Instruments, S.A., Athens, Greece). A LINXEYE XE high-resolution energy-dispersive detector was mounted on the XRD instrument. The possible interactions between the SBA-15 and the adsorbed TEO molecules were investigated via FTIR spectroscopy measurements in both SBA-15 and TEO@SBA-15 nanohybrid materials using a JASCO FT/IR-6000 Fourier-Τransform spectrometer provided by Interlab, S.A. Company, located in Athens, Greece. Pure SBA-15 and TEO@SBA-15 nanohybrid were also characterized gravimetrically to estimate the total TEO load on these materials. Such measurements were carried out using a Perkin Elmer Pyris Diamond TGA/DTA instrument provided by the last mentioned Interlab, S.A. company. These materials were furthermore characterized by the Differential Scanning Calorimetry (DSC) instrument DSC214 Polyma provided by the manufacturer NETZSCH, located in Selb, Germany.

### 2.6. Physicochemical Characterization of LDPE/xSBA-15 Films and LDPE/xTEO@SBA-15 Films

The experimental conditions used for the physicochemical characterization of all LSPE/xSBA-15 and LDPE/xTEO@SBA-15 films are described in detail in previous recent publications [21,23,24]. Briefly, a Bruker XRD D8 Advance diffractometer, Bruker GmbH, Mannheim, Germany was employed to carry out XRD analysis to all films i.e., LDPE/xSBA-15, LDPE/xTEO@SBA-15, and the pure LDPE films, to define the crystal structures of the resulted materials. The interactions between the incorporated SBA-15 and TEO@SBA-15 nanostructures with the LDPE polymeric matrix were studied using an FT/IR-6000 JASCO Fourier-transform spectrometer, JASCO company, Easton, MD, USA. 

### 2.7. Mechanical and Thermomechanical Properties of LDPE/xSBA-15 and LDPE/xTEO@SBA-15 Films

Following the methodology proposed in the literature and according to the ASTM D638 standard reported in such references [21,23,24], all films were studied for their tensile properties. For such measurements, a Simantzü AG-X 5kNt instrument was used provided by the Simantzu. Asteriadis, S.A. company which is located in Athens Greece. Film tension behavior was also investigated by employing a DMA Q800, TA Instruments dynamic analyzer, New Castle, DE, USA. All measurements were carried out with a temperature ramp of 5 °C/min, a temperature range of 30 °C to 120 °C, and a frequency of 1 Hz. The storage modulus (E’) and the loss factor (tan δ) were determined during such measurements.

### 2.8. SEM Images

A JEOL JSM 6510-LV SEM instrument (JEOL GmbH, Freising, Germany) was employed to study the surface morphology of the obtained films. Images were achieved by setting up an acceleration voltage of 20 kV.

### 2.9. Water Vapor Transmission Rate (WVTR) and Water Vapor Diffusion Coefficient Calculation of LDPE/xSBA-15 and LDPE/xTEO@SBA-15 Films

The method proposed by the ASTM E96/E 96M-05 standard was followed during this work to measure WVTR values for all the obtained films. A handmade apparatus reported in the literature [35,36] was used, and the water vapor diffusion coefficient values (D_wv_) were estimated according to the model described in detail recently [37].

### 2.10. Oxygen Transmission Rate and Oxygen Permeability Calculation of LDPE/xSBA-15 and LDPE/xTEO@SBA-15 Films

An O.P.A. 8001 oxygen permeation analyzer instrument provided by the Systech Illinois Instruments Co. company (Johnsburg, IL, USA) was employed to estimate the Oxygen Transmission Rate (O.T.R.) values of all obtained LDPE/xSBA-15, LDPE/xTEO@SBA-15, and “blank” LDPE films. Such measurements were carried out according to the ASTM D 3985 method at a temperature of 23 °C, and relative humidity of 0% RH. Oxygen permeability coefficient values (P_O2_) were estimated using the O.T.R. measurements according to the model proposed recently in detail [37] and by using the dimensionally derived Equation (1):

(1) $$P_{O2}=\mathrm{OTR}\times \Delta x$$

where OTR is the oxygen transmission rate through the film (cm^3^ O_2_ STP·cm^−2^ film area·s^−1^) and Δx the mean film thickness (cm).

### 2.11. TEO Desorption Kinetics and Calculation of TEO Release Diffusion Coefficient (D_TEO_)

Predictive modeling of release from packaging materials into food products is an advanced tool for active packaging materials containing EOs [38]. For all the obtained LDPE/xTEO@SBA-15 active films TEO release experiments were conducted by employing an AXIS AS-60 moisture analyzer, AXIS Sp. z o.o., 80-125 Gdansk, Poland, according to the methodology described recently [24]. Films with 10 cm diameter and approximately 300 to 500 mg were placed inside the chamber of the moisture analyzer and the mass loss was recorded at 70 °C since the mass remained constant. From these experiments, the wt.% total TEO content release was calculated while the diffusion coefficient (D_TEO_) for the release of TEO was calculated by using the following Equation (2):

(2) $$\frac{m_{t}}{m_{\infty }}=\sqrt{4\frac{D\cdot t}{\pi \cdot l^{2}}}$$

where m_t_ and m_∞_ are the amount of TEO released from the film after time t and after the equilibrium time t_eq_→∞, respectively, D is the diffusion coefficient for the TEO release process, and l is the average film thickness.

The linearization of Equation (2) leads to the slightly modified Equation (3):

(3) $${\left(\frac{m_{t}}{m_{\infty }}\right)}^{2}=4\frac{D\times t}{\pi \times l^{2}}$$

By employing the pseudo-second-order sorption mechanism model [34], we calculated the desorption rate constant k_2_ and predicted the maximum TEO desorbed amount when the system reached the equilibrium stage (q_e_). The pseudo-second-order kinetic equation is as follows:

(4) $$q_{t}=\frac{q_{e}^{2}\times k_{2}\times t}{q_{e}\times k_{2}\times t+1}$$

where q_t_ = m_t_/m_0_ and q_e_ = 1.

### 2.12. Antioxidant Activity of LDPE/xTEO@SBA-15 Films with DPPH (2,2-diphenyl-1-picrylhydrazyl) Assay

#### 2.12.1. Preparation of DPPH Free Radical Standard Solutions

For the total antioxidant activity measurements 250 mL of a standard DPPH ethanolic solution with 2.16 mM (mmol/L) was prepared according to the method proposed by Krishnanad et al. [39]. The obtained solution was refrigerated for 2 h before use. Once the free radical was stable, appropriate dilutions were carried out to establish the calibration curve. 

#### 2.12.2. Preparation of DPPH Free Radical Calibration Curve

A calibration curve was developed using a Jasco V-530 UV/VIS Spectrometer instrument. Five DPPH ethanolic solutions with five different concentrations in the range of 0–60 mg/L (ppm) were prepared. The absorbance values at λ_max_ = 517 nm [40] and the concentration values of the DPPH^•^ of the ethanolic solutions were used as a couple of coordinates to obtain a linear curve. The linear equation, which was derived via the numerical fitting of this experimental curve, was sequentially employed to determine the concentration values of the remaining DPPH^•^ roots using the UV absorbance experimental measurements and following the procedure that was reported previously [41]. 

#### 2.12.3. Total Antioxidant Activity of LDPE/xTEO@SBA-15 Films

The antioxidant activity of all obtained LDPE/xTEO@SBA-15 active films was measured using the 2,2-diphenyl-1-picrylhydrazyl radical (DPPH) assay, as described previously [35,36,37]. Briefly, in 2.8 mL of 30 ppm DPPH ethanolic solution, 0.2 mL of CH_3_COONax3H_2_O buffer solution, and approximately 2 mg of each LDPE/xTEO@SBA-15 active film was added, and the absorbance at 517 nm was recorded as a function of time every day for 1 weak total time. As a blank sample, the absorbance of 4 mL of 30 ppm DPPH ethanolic solution without the addition of any film was also recorded as a function of time. The % antioxidant activity was calculated by using Equation (5)

(5) $$\%{\;\mathrm{DPPH}}^{•}\;\mathrm{scavenged}\;\mathrm{at}\;\mathrm{steady}\;\mathrm{state}\;=\frac{A_{0}^{517}-A_{sample}^{517}}{A_{0}^{517}}\times 100$$

#### 2.12.4. Estimation of EC50 (Concentration Required to Obtain 50% Antioxidant Effect) Antioxidant Activity of LDPE/xTEO@SBA-15 Films

Five different species of each kind of film, weighing from 1 to 5 mg, respectively, were separately added to five different solutions, which contained 2.8 mL of 30 ppm DPPH ethanolic solution, and 0.2 mL of CH_3_COONa•3H_2_O buffer solution. After a 1-h timepass and assuming that steady-state conditions were reached, UV-vis measurements were carried out to determine the absorbance of each solution at a 517 nm wavelength. The antioxidant activity of each sample was estimated indirectly using Equation (4). The higher the percentage of DPPH^•^ scavenged at steady state, the higher the percentage of antioxidant activity of the antioxidant material [42]. Next, the calculated values of antioxidant activity of each film were plotted as a function of film quantity added in the DPPH solution, and the linear equation from the obtained experimental point plot was developed. From the obtained linear equation of each coating, the EC_50_ value (i.e., the quantity of film exhibiting 50% antioxidant activity) was estimated.

### 2.13. Packaging Test of Fresh Pork Fillets Wrapped with LDPE/TEO@SBA-15 Active Film

**Figure 2:**
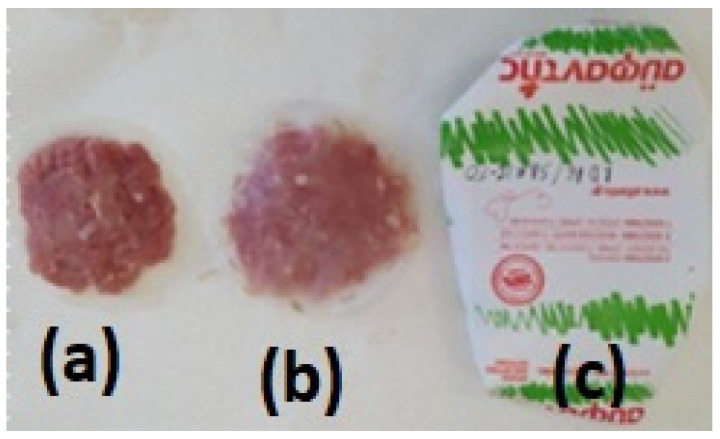
(**a**) Raw minced pork meat; (**b**) minced pork meat between two LDPE/TEO@SBA-15 active films; (**c**) minced pork meat wrapped with commercial packaging paper.

Fresh minced pork meat was provided by a local meat processing plant (Aifantis Company-Aifantis Group—Head Quarters, Acheloos Bridge, Agrinio, Greece 30100) and immediately transported to the laboratory. For the packaging test, LDPE and LDPE/10TEO@SBA-15 films with approx. diameter of 10 cm and approx. thickness of 0.10 mm were used. For the packaging, the packaging paper from the local meat processing plant was used. From this packaging paper, the internal side packaging film was removed prior to use. Approximately 25 g of fresh pork minced meat were wrapped inside two LDPE or LDPE/10TEO@SBA-15 films and then wrapped with the paper package used by the processing plant (see Figure 2).

#### 2.13.1. Lipid Oxidation of Minced Pork Meat with Thiobarbituric-Acid-Reactive Substances

The thiobarbituric-acid-reactive substances (TBARS) values of the wrapped pork fillets were determined using the method of Tarladgis et al. [43]. The methodology for determining the TBARS values of packaged fresh pork fillets was described in detail recently [21,23]. TBARS value analyses were carried out every 2 days up to 8 days of storage at 4 ± 1 °C.

#### 2.13.2. Heme Iron Content

The heme iron content of the wrapped with pure LDPE film and LDPE/15TEO@SBA-15 active film fresh minced pork meat was determined according to the method reported by Clark et al. [44], and as described in detail recently [23,24]. Heme iron content analyses were carried out every 2 days up to 8 days of storage at 4 ± 1 °C.

#### 2.13.3. pH Values of Minced Pork Meat

The pH values of the pork minced meat coatings were measured using a portable pH meter fitted with a penetration electrode and a temperature sensor (pH-Star, Matthäus GmbH, Poettmes, Germany). Prior to each set of measurements, the pH meter was calibrated using pH standard solutions of 4.0 and 7.0 and temperature-adjusted to match the meat coating temperature of 4 °C. The entire study was conducted in triplicate, and for each treatment group, ten separate pH readings were taken to ensure accuracy and reliability, as per the methods [45]. Overall, all coatings displayed an increase in pH over the 9-day analysis period.

#### 2.13.4. Total Variable Counts (TVCs) of Pork Fillets

The TVCs were monitored every 2 days up to 8 days of storage at 4 ± 1 °C. Ten grams of pork fillet were removed aseptically from each packaging treatment using a spoon, transferred to a stomacher bag (Seward Medical, Worthing, West Sussex, UK) containing 90 mL of sterile buffered peptone water (ΒPW, NCM0015A, Heywood, BL97JJ, UK) (0.1 g/100 mL of distilled water), and homogenized using a stomacher (LAB Blender 400, Seward Medical, UK) for 90 s at room temperature. For the microbial enumeration, 0.1 mL of serial dilutions (1:10 diluents, buffered peptone water) of pork meat homogenates was spread on the surface of plate count agar (PCA, NCM0010A, Heywood, UK). The TVCs were determined every 2 days up to 8 days of storage at 4 ± 1 °C after incubation of each plate for 2 days at 30 °C [46].

#### 2.13.5. Sensory Analysis Testing of Pork Fillets

The sensory properties of pork fillets were scaled from 0 (for the least liked sample) to 5 (for the most liked sample) points by seven experienced members of the Food Science and Technology Department. On each sampling day, color, odor, and cohesion were evaluated [47].

### 2.14. Statistical Analysis

In this study the IBM, SPSS 2.5, software was used for statistical analysis. The non-parametric Kruskal-Wallis test was employed to determine the significance of the mean value difference at the 5% level (*p* < 0.05). Pearson’s bivariate correlation, ranging from −1 to +1, was used to estimate the correlation between heme iron and TBARS. This was conducted with a confidence level of *p* < 0.05.

## 3. Results

### 3.1. Physicochemical Characterization of SBA-15 and TEO@SBA-15 Nanohybrid

**Figure 3:**
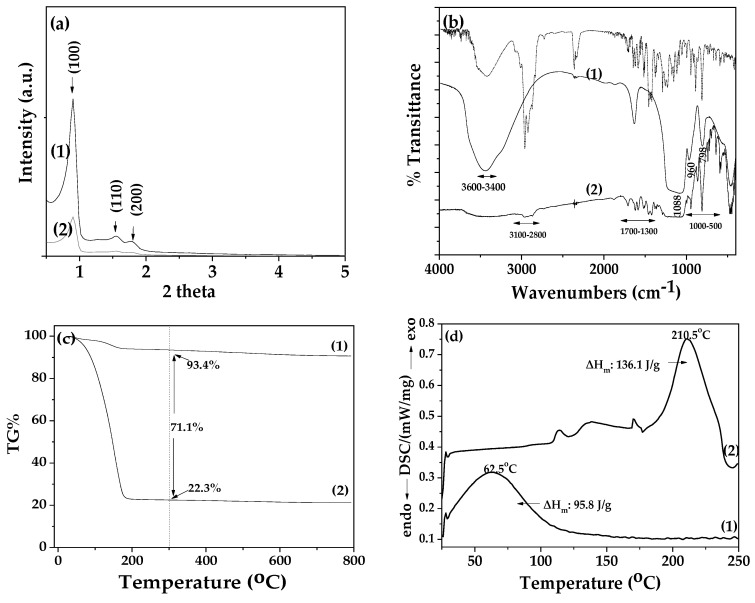
(**a**) X-ray Diffraction (XRD) plots of (1) pure SBA-15 and (2) modified TΕO@SBA-15 nanohybrid in the range of 0.5°–5° 2theta, (**b**) Fourier Transform Infrared (FTIR) plots of (1) pure SBA-15, (2) modified TEO@SBA-15 nanohybrid and with dash dot line pure TEO at the range of 400–4000 cm^−1^, (**c**) Thermogravimetric Analysis (TGA) plots of (1) pure SBA-15 and (2) modified TEO@SBA-15 nanohybrid in the range of 25 to 800 °C temperature and (**d**) Differential Scanning Calorimetry (DSC) plots of (1) pure SBA-15 and (2) modified TEO@SBA-15 nanohybrid (blue line) in the range of 0 to 250 °C temperature.

In Figure 3, the XRD, FTIR, TG, and DSC plots of SBA-15 and TEO@SBA-15 nanohybrid are shown.

The XRD plot of pure SBA-15 (see Figure 3a line 1) exhibits a single high-intensity peak (100) at 2Ɵ value of 0.96°, followed by two additional smaller peaks, at (110) and (200), at 2Ɵ lower than 2°, which confirms the formation of a hexagonal lattice of p6mm symmetry [26,30,34,48]. On the contrary, in the XRD plot of the modified TEO@SBA-15 nanohybrid (see Figure 3a line 2) the adsorption of TEO molecules gave rise to a strong decrease in the intensity of the characteristic reflections of SBA-15. This should be attributed to the pore-filling effects due to the adsorption of TEO molecules in the inner space of SBA-15’s hexagonal structure, which can reduce the scattering contrast between the pores and the silica walls [30].

In the FTIR plot of pure SBA-15 (see Figure 3b, line 1), the stretching vibrations observed in the range of 3600–3400 cm^−1^ are attributed to the hydrogen-bonded silanol groups of SBA-15 [26,30,34,49]. The band at 1088 cm^−1^ is assigned to the n (Si–O–Si) asymmetric vibrations of Si–O–Si groups of SBA-15, and the band at 798 cm^−1^ is assigned to the symmetric vibrations of the Si–O groups of SBA-15, while the band at 960 cm^−1^ is attributed to the vibration of Si–OH groups of SBA-15 [49]. In the FTIR plot of the modified TEO@SBA-15 nanohybrid (see Figure 3b, line 2), the same reflections of SBA-15 are observed with much lower intensity than in the pure SBA-15 FTIR plot. In addition, in the FTIR plot of the TEO@SBA-15 nanohybrid, the characteristic bands of TEO molecules are observed. For comparison, in the upper part of Figure 3b, the FTIR plot of TEO is shown with a dash dot line. The bands at around 3100–3000 cm^−1^ are ascribed to the stretching vibrations of aromatic and alkenic groups of TEO molecules, the bands at 2958 and at 2868 cm^−1^ are assigned to the stretching mode of C-H groups, and the bands between 1500 cm^−1^ and 1300 cm^−1^ are assigned to the bending of C-H on the C-O-H group and the bending of aliphatic CH_2_ groups implying the loading of TEO molecules on the SBA-15 surface [20,24]. In addition, comparing the FTIR plot of TEO@SBA-15 nanohybrid with that of pure SBA-15, it is observed that there is a broadening of the stretching vibrations in the range of 3600–3400 cm^−1^ which are attributed to the hydrogen-bonded silanol groups of SBA-15. This fact suggests a kind of bonding/relaxation of TEO molecules with the hydroxyl groups of SBA-15. 

In the TG plot of pure SBA-15 (see Figure 3c line 1) one small mass loss step is observed starting at before 100 °C and ending at before 200 °C. This mass loss is attributed to the water molecules’ desorption [30,34]. In the TG plot of the modified TEO@SBA-15 nanohybrid (see Figure 3c line 2) the mass loss step is huge. It begins above 100 °C and ends before 300 °C and is attributed to the TEO molecules’ adsorption mass loss step [30]. Above 300 °C since 800 °C, the mass remains constant for both pure SBA-15 and modified TEO@SBA-15 nanohybrid. Thus, at the temperature of 300 °C it has been calculated that the final percentage mass loss for SBA-15 is equal to 22.3%, and for modified TEO@SBA-15 nanohybrid equal to 93.4%. So, it is calculated that the total amount of TEO adsorbed in SBA-15 is equal to 71.1%. This result means that SBA-15 adsorbs a high amount of TEO, much higher than nanocarriers such as activated carbon, natural zeolite, and halloysite nanoclays recently reported [21,23,24]. This result is not only because of the high surface area of SBA-15 but also due to its tunable mesopore diameter of between 5 and 15 nm. This result was also combined with the results of XRD and FTIR discussed hereabove, where it was shown that the reflections of SBA-15 decreased, as shown in both XRD and FTIR plots of TEO@SBA-15. The fact that SBA-15 can adsorb much higher amounts of TEO than activated carbon, natural zeolite amorphous silica (SiO_2_), and nanoclays validates its use as a novel nanocarrier for controlling the release of EOs in food packaging applications. Even though SBA-15 is more expensive than amorphous silica, its ability to adsorb much more of EO’s content makes it a potential polymer nanofiller for active food packaging control release applications [27]. For example, considering that the final TEO %wt. content adsorbed in SBA-15 is approx. 71.1% of the 10 %wt. addition of TEO@SBA-15 nanohybrid in the polymer matrix corresponds to approx. 3 %wt. SBA-15 addition in the obtained film.

In the DSC plot of pure SBA-15 (see Figure 3d, line 1), the exothermal step with a peak at 62.5 °C and a ΔH equal to 95.8 J/g is attributed to the desorption of water molecules from the mesoporous of SBA-15. In the DSC plot of modified TEO@SBA-15 (see Figure 3d, line 2), there is a main large exothermic peak at 210 °C, which corresponds to the desorption of TEO molecules from the tunable mesoporous of SBA-15 [21,23,24]. This exothermic peak of TEO molecules desorption combines with the FTIR results discussed here above and suggests partial bonding/relaxation of TEO molecules with the hydroxyl groups of SBA-15.

### 3.2. Physicochemical Characterization of LDPE/xSBA-15 and LDPE/xTEO@SBA-15 Films

**Figure 4:**
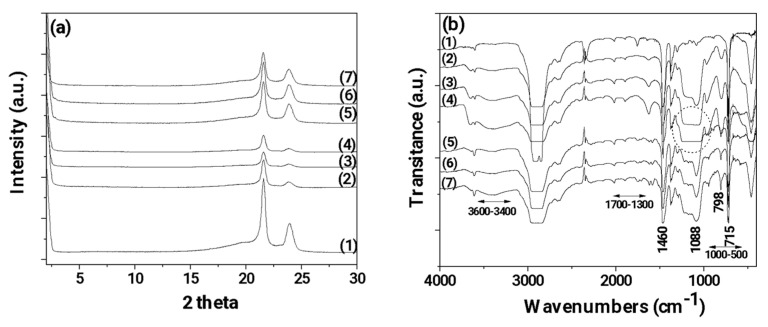
(**a**) X-ray diffraction (XRD) plots of pure LDPE and all LDPE/xSBA-15 and LDPE/TEO@SBA-15 films; (**b**) Fourier Transform Infrared (FTIR) plots of pure LDPE and all LDPE/xSBA-15 and LDPE/TEO@SBA-15 films. (1) pure LDPE, (2) LDPE/5SBA-15, (3) LDPE/10SBA-15, (4) LDPE/15SBA-15, (5) LDPE/5TEO@SBA-15, (6) LDPE/10TEO@SBA-15, (7) LDPE/15TEO@SBA-15.

In Figure 3, the XRD (Figure 4a) and FTIR (Figure 4b) plots of all LDPE/xSBA-15 and LDPE/TEO@SBA-15 films as well as pure LDPE film are shown for comparison.

In all XRD plots (see Figure 4a) of the obtained LDPE/xSBA-15 and LDPE/TEO@SBA-15 films as well as pure LDPE film the characteristic peaks of LDPE crystal phase at Bragg angles 2θ = 21.5° and 23.75° are observed. It is also observed that by the addition of both SBA-15 nanostructure and TEO@SBA-15 nanohybrid the LDPE’s peaks decreased. In the case of all LDPE/xSBA-15 films, the decrease in LDPE’s characteristic peaks is higher than in the case of all LDPE/xTEO@SBA-15 films. This decrease is more pronounced in the case of LDPE/10SBA-15 and LDPE/15SBA-15 films. This is an indication of the higher dispersion achieved for TEO@SBA-15 nanohybrid in the LDPE matrix than for pure SBA-15 [24]. 

In all cases of the FTIR plots (see Figure 4b) of all obtained LDPE/xSBA-15 and LDPE/TEO@SBA-15 films as well as pure LDPE film the characteristic peaks of LDPE are obtained. The bands at 1460 and 715 cm^−1^ are assigned to the asymmetric stretching of the CH_3_ group, the group wagging of the CH_2_ group, and the group rocking of the CH_2_ group of the LDPE. In all LDPE/xSBA-15 and LDPE/xTEO@SBA-15 films, the characteristic peaks of LDPE are decreased and the characteristic peaks of SBA-15 at 3600–3400 cm^−1^, at 1088 cm^−1^, and 798 cm^−1^ are observed. In advance in the case of LDPE/xTEO@SBA-15 films the extra small peaks in the range of 1700–1300 cm^−1^ and the range of 1000–500 cm^−1^ prove the presence of TEO molecules. No shift peak of LDPE’s characteristic peaks is observed implying no chemical bonding between the LDPE matrix and SBA-15 or TEO@SBA-15 chemical groups [50]. With a more careful glance, it is observed that the characteristic peak of SBA-15 at 1088 cm^−1^ is much higher in the case of all LDPE/xSBA-15 (especially for LDPE/10SBA-15 and LDPE/15SBA-15 see peaks depicted with the dot line cycle) films than in the case of LDPE/TEO@SBA films. This fact combines with the XRD plots results and recent reports and suggests that the modified and more hydrophobic TEO@SBA-15 nanohybrid achieves higher dispersion in the LDPE matrix than pure SBA-15 [21,23,24].

### 3.3. Mechanical and Thermomechanical Properties of LDPE/xSBA-15 and LDPE/xTEO@SBA-15 Films

The calculated Elastic Modulus (E), ultimate strength (σ_uts_), and elongation at break (%ε) values for all obtained LDPE/xSBA-15 and LDPE/xTEO@SBA-15 films as well as for pure LDPE film are listed in Table 1 for comparison.

As it is obtained from the values of Elastic Modulus (E), ultimate strength (σ_uts_), and elongation at break (%ε) calculated and listed in Table 1, the addition of pure SBA-15 in the LDPE matrix increases the stress values and decreases both ultimate strength and % elongation at break values. This is typical behavior when rigid inorganic particles such as SBA-15 are added to the LDPE matrix [23,50,51]. On the contrary, when the modified TEO@SBA-15 nanohybrid is added to the LDPE matrix, the stress values increase while the ultimate strength and % elongation at break values remain statistically constant. This fact suggests the higher compatibility of modified TEO@SBA-15 nanohybrid with LDPE polymer matrix due to its hydrophobic modification via adsorption of TEO molecules, and combined with the results of XRD and FTIR discussed hereabove, and suggested higher dispersion of TEO@SBA-15 in the LDPE matrix than pure SBA-15.

**Figure 5:**
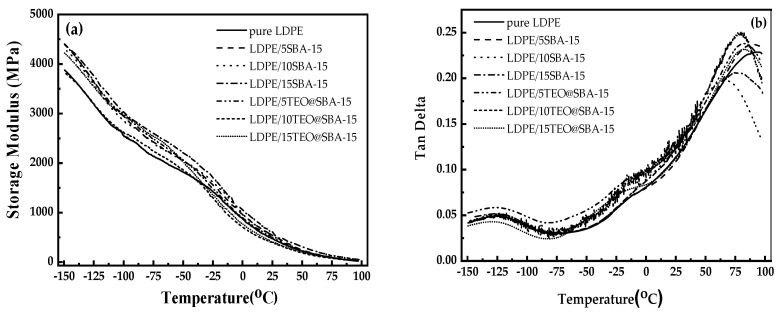
(**a**) Storage Modulus curves and (**b**) Tan Delta curves of pure LDPE (1), LDPE/5SBA-15 (2), LDPE/10SBA-15 (3), LDPE/15SBA-15 (4), LDPE/5TEO@SBA-15 (5), LDPE/10TEO@SBA-15 (6), and LDPE/15TEO@SBA-15 (7).

Figure 5a presents the data of the storage modulus as a function of temperature from sub-glass transition temperature (T  <  T_g_) up to 100 °C, and Figure 5b presents the tan δ values as a function of temperature for all LDPE/xSBA-15, LDPE/xTTEO@SBA-15 films, as well as pure LDPE film.

The curves of storage modulus (see Figure 5a) versus temperature, reveal the difference between the pure polymer matrix and nanocomposite materials. The samples do not show a considerable variation in their elasticity, although the highest values are observed for the LDPE samples mixed with SBA-15. The values of the Storage Modulus range from 3793 MPa to 4393 MPa. The curves of all the compositions appear in the following regions: (a) the glassy region (T < −120 °C) (b) the transition region (−120 °C < T ≤ −100 °C), (c) the rubbery region (−100 °C < T ≤ −65 °C), and (d) the secondary transition region (−65 °C < T ≤ −20 °C).

The value of tan δ (see Figure 5b) is calculated from the ratio of E”/E’ and shows the damping behavior (mechanical loss of energy) of the samples when increasing the SBA-15 content. In general, the decrease in tan δ with increasing SBA-15 content indicates the reinforcement ability of the integration of SBA-15. Rigid polymers present higher storage modulus values and correspondingly lower loss modulus values, thus reducing the intensity of t_anδ_ peaks. In softer materials, where the viscous part dominates, higher energy loss is observed, resulting in a higher intensity of tan δ peaks. Hence, the highest peak in Figure 4b belongs to the LDPE nanocomposite with the lower SBA-15 content (5%), and the lowest peak, to the LDPE nanocomposite with the highest SBA-15 content (15%).

### 3.4. Morphological Characterization and Comparison of LDPE/xSBA-15 and LDPE/xTEO@SBA-15 Films Using an SEM Instrument

The surface morphology of the LDPE matrix and the hybrid nanocomposite films of LDPE/SBA-15 and LDPE/TEO@SBA-15 were investigated using an SEM instrument, and the results confirmed that the SBA-15 and hybrid nanostructure TEO@SBA-15 were homogeneously dispersed in the polymer matrix.

**Figure 6:**
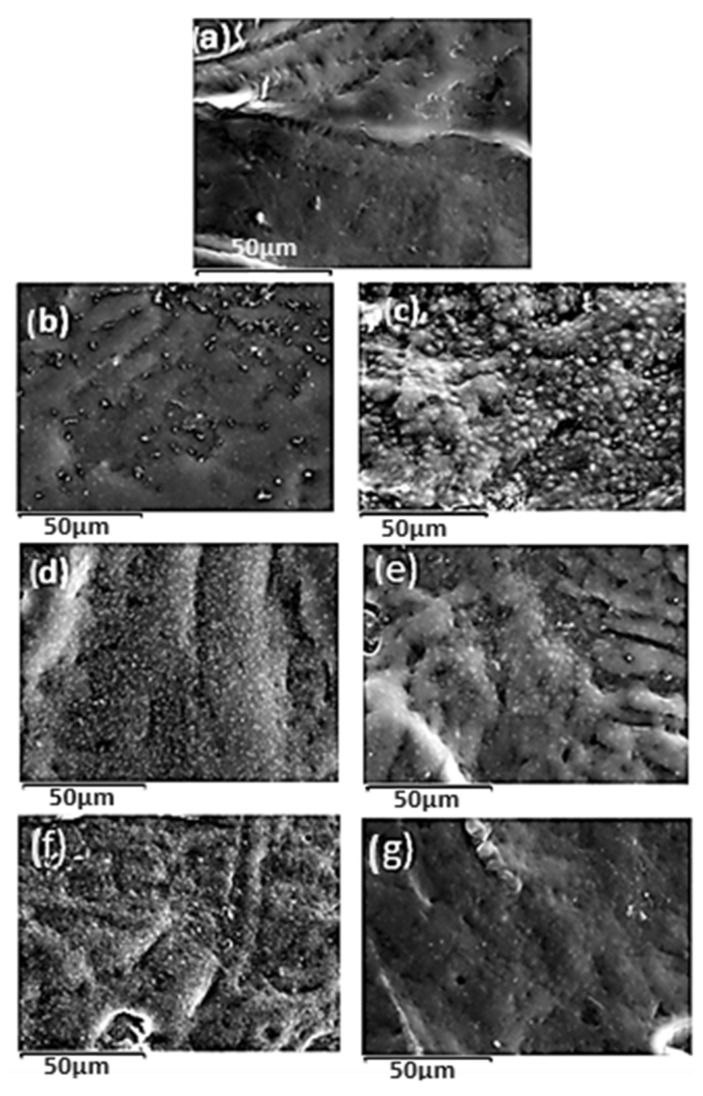
SEM micrographs of (**a**) LDPE (**b**) LDPE/5SBA-15 (**c**) LDPE/5TEO@SBA-15 (**d**) LDPE/10SBA-15 (**e**) LDPE/10TEO@SBA-15 (**f**) LDPE/15SBA-15 (**g**) LDPE/15TEO@SBA-15.

The SEM images (surface) in Figure 6a exhibit the expected homogeneous structure of the pristine polymer matrix (LDPE).

Surface images of LDPE/xSBA-15 and LDPE/xTEO@SBA-15 films, where x = 5, 10, and 15 wt.%, are presented in Figure 6b,d,f and Figure 6c,e,g, respectively. Based on the SEM studies of the surface morphology, it should be mentioned that a significant difference is observed when the TEO@SBA-15 hybrid nanostructure is integrated into the polymer matrix since better interfacial adhesion and homogenous dispersion is evident when compared with the respective nanocomposite film with pure SBA-15.

### 3.5. Water-Oxygen Barrier Properties of LDPE/xSBA-15 and LDPE/xTEO@SBA-15 Films

In Table 2 the obtained water vapor transmission rate (WVTR), and oxygen transmission rate (OTR) mean values of all tested LDPE/XSBA-15 and LDPE/XTEO@SBA-15 films as well as for pure LDPE film are listed for comparison. From these values, the water vapor diffusion coefficient (D_wv_) values and the oxygen permeability (P_O2_) values were calculated and listed in Table 2 for comparison too.

As is observed from the calculated water vapor diffusion coefficient (D_wv_) and the oxygen permeability (P_O2_) values listed in Table 2 both pure SBA-15 and modified TEO@SBA-15 nanofillers achieved to increase both the water and oxygen barrier of obtained films. According to the literature when such SBA-15 nanofiller is dispersed in a polymer media hexagonal arrays of cylindrical mesopores parallel to the polymer substrate are obtained with a (h00) preferred orientation [52,53,54]. In addition, as was shown in the SEM images section both pure SBA-15 and modified TEO@SBA-15 nanofillers dispersed homogeneously in the LDPE matrix and thus succeeded in reducing the free volume available for the water/oxygen molecules permeation [55]. This may be the explanation for the increase of both the water and oxygen barrier of obtained films. Considering that such films will be used as active packaging films the optimum one is that with code name LDPE/10TEO@SBA-15 which achieves 55% and 46% higher water and oxygen barrier than pure LDPE film correspondingly.

### 3.6. ΤΕO Control Release Kinetics from LDPE/xTEO@SBA-15 Films

By using the Equations (2) and (3) the wt.% TEO total released amount (%m_TEO_), the diffusion coefficient of TEO released, the released equilibrium amount of TEO (q_e_), and the desorption rate constant value (K_2_) for all studied LDPE/xTEO@SBA-15 films were calculated and are listed in Table 3 for comparison.

As it was expected the calculated values of the wt.% TEO total released amount (m_TEO_) and the released equilibrium amount of TEO (q_e_) increases with the increase of wt.% TEO loaded on LDPE/xTEO@SBA-15 active films in accordance with recent reports [21,23,24]. On the other hand, the calculated values of the diffusion coefficient of TEO (D_TEO_) released from the film and the desorption rate constant (K_2_) decrease as the wt.% TEO loaded on LDPE/xTEO@SBA-15 active films increases. This means that as the TEO loaded amount on the obtained LDPE/xTEO@SBA-15 active films increases the release rate accelerates. This phenomenon could be an indication that the increase of the SBA-15 loaded amount in the LDPE matrix decreases the diffusion paths that are available for TEO molecules to release. Comparing the D_TEO_ calculated for such LDPE/xTEO@SBA-15 and the diffusion coefficient values calculated recently for thymol release from LDPE/xTO@AC (TO: thymol, AC: activated carbon) it is obtained that the D_TEO_ values for LDPE/xTEO@SBA-15 are on order of magnitude higher [24]. This means that SBA-15 is a very promising nanocarrier for the controlled release of such EOs in active food packaging films as it achieves high adsorption amounts of EOs and releases them at high rates. 

### 3.7. Antioxidant Activity of LDPE/xTEO@SBA-15 Films

**Figure 7:**
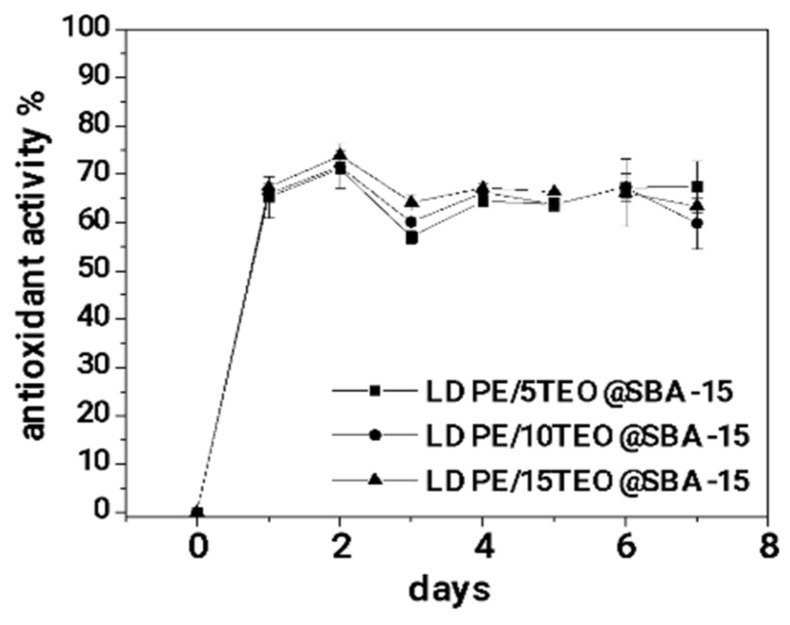
The % total antioxidant activity of all obtained LDPE/xTEO@SBA-15 active films as a function of time.

In Figure 7, the calculated values for % antioxidant activity of all obtained LDPE/xTEO@SBA-15 active films as a function of time (for seven days) are plotted. 

As it is observed in Figure 7 all the obtained LDPE/xTEO@SBA-15 active films achieve a high % total antioxidant activity in the range of 65–70%. In addition, the % total antioxidant activity for all obtained LDPE/xTEO@SBA-15 active films was achieved after 48 h and remained almost constant for seven days (one weak). 

To figure out better the antioxidant activity of all obtained LDPE/xTEO@SBA-15 active films the % effective concentration (EC_50_) values were calculated and are listed in Table 3 for comparison. As it is obtained from the calculated EC_50_ values the most active film is the LDPE/10TEO@SBA-15 film with the lowest EC_50_ value equal to 4.57. This result seems controversial because LDPE/10TEO@SBA-15 was not the film with the higher TEO amount load. An explanation for this result could be the release rate of TEO from LDPE/10TEO@SBA-15. As it was obtained from Table 3 LDPE/10TEO@SBA-15 film was loaded with a lower amount of TEO than LDPE/15TEO@SBA-15 film but exhibited higher diffusion coefficient (D_TEO_) and release constant rate (k_2_) values than LDPE/15TEO@SBA-15 film. This means that the TEO release was easier in the case of LDPE/10TEO@SBA-15.

### 3.8. Lipid Oxidation of Pork Fillets

The calculated TBARS and heme iron content values of the low-fat pork fillets wrapped with pure LDPE, LDPE/10SBA-15, and LDPE/10TEO@SBA-15 films are shown in Table 4 for comparison.

As it is obtained the listed TBARS values from 0 to 8th day align with those reported in recent similar studies [21,23,24]. In addition, it is obtained that LDPE/10TEO@SBA-15 active film succeeded in reducing obtained TBARS values during the 8 days of storage in comparison to the relevant TBARS values of the pure LDPE film. Indeed, the results observed on day 8 were statistically significant at *p* < 0.05 level (See Table S1). Regarding the listed in Table 4 calculated values of heme iron for minced pork meat wrapped with pure LDPE film and LDPE/10TEO@SBA-15 active film, a significant difference was observed on days 2, 4, and 8 (See Table S2). Moreover, heme iron values remain higher for minced pork meat wrapped with LDPE/10TEO@SBA-15 active film than the hem iron values of minced pork meat wrapped with pure LDPE film. The correlation between heme iron and lipid oxidation by day 8 was −0.930, indicating a strong, statistically significant negative relationship (See Table S3). This suggests that as TBARS decreases, heme iron increases. Overall results from TBARS and heme iron values suggest that LDPE/10TEO@SBA-15 active film succeeds in accelerating minced pork meat lipid oxidation and keeps it in a higher nutritional condition.

### 3.9. pH Analysis of Minced Pork Meat

In Table 5, the obtained pH values for all treatments used for the 8-day examined period are listed for comparison.

As is observed in Table 5 pH values of minced pork meat wrapped with pure LDPE and minced pork meat wrapped with LDPE/10TEO@SBA-15 active film decrease as the storage period increases implying the growth of pathogenic bacteria during the storage period. However, the decrease in pH observed in both treatments does show a statistically significant difference (see Table S2).

### 3.10. Microbiological Changes of Minced Pork Meat

The total viable count (TVC) of bacteria is an important microbiology indicator for the sanitary quality and safety evaluation of meat [56,57]. It is the quantitative sanitary standard to identify the process conditions and contamination degree of meat [47,57]. The TVC values are given a direct correlation with the population of food microorganisms such as bacteria, yeasts, and molds in a food sample capable of forming visible colonies. Most microorganisms present in pork minced meat, either as a part of its natural microflora or as the result of cross-contamination from other sources, are mostly aerobic microorganisms and their population is an indicator of product microbiological quality [47]. Table 6 lists the changes in calculated TVC values of minced pork meat as a function of film used and storage time.

As is observed in Table 6 and during the 6 days of storage TVC values of minced pork meat wrapped with LDPE/10TEO@SBA-15 actively increased at a lower rate than the TVC values of minced pork meat wrapped with pure LDPE film. By day 6, the LDPE/10TEO@SBA-15 active film observed a count of 7.11 log CFU/g, indicating a difference of 0.25 log as compared to 7.36 log CFU/g of pure LDPE, which is statistically significant (See Table S5). In other words, LDPE/10TEO@SBA-15 active film succeeded in accelerating the TVC growth rate of minced pork meat as compared to pure LDPE film.

### 3.11. Sensory Evaluation of Pork Fillets

Sensory properties such as color, odor, and cohesion are major factors for consumers to accept and buy a meat food product [45,58,59]. Color is an important quality attribute of fresh and processed meat. The main meat pigments are myoglobin (or myoglobin) and hemoglobin (hemoglobin). Myoglobin predominates in-ground and well-mixed muscle tissues [60,61]. Off odors in spoiled pork meat could be related to compounds originating from the growth of some microorganisms, or chemical compounds such as ammonia or amines resulting from protein breakdown and also ketones and aldehydes resulting from lipid oxidation [47,49,50,61]. The sensory evaluation results of the present study are displayed in Table 7.

As it is observed in Table 7 the minced pork meat wrapped with LDPE/10TEO@SBA-15 active film succeeded in providing higher sensory characteristics in color, odor, and cohesion as compared to the sensory characteristics of pork meat wrapped with pure LDPE film. More specifically minced meat wrapped with pure LDPE film has odor and cohesion values lower than the minimum acceptable 3 value after 6 days of storage.

In the statistical analysis of sensory evaluation comparing LDPE and LDPE/10TEO@SBA-15 treatments. It is observed that findings include statistically significant differences in odor and color on Day 6. Additionally, cohesion showed significant differences on Days 2, 4, and 6 indicating that the LDPE/10TEO@SBA-15 active film can positively impact way on the sample over time.

## 4. Conclusions

According to the results of the current study, SBA-15 porous material is a potential novel TEO nanocarrier for the development of a hybrid TEO@SBA-15 nanostructure. XRD characterization of this nanostructure indicated the adsorption of the TEO inside pores while the FTIR measurements have shown a bonding/relaxation of the TEO molecules with hydroxyl functional groups on the SBA-15 pore surface. The latter was confirmed by DSC measurements which further support furthermore the hypothesis of control release and not bulk release of the TEO. TG analysis resulted in an overall TEO loading of 71.1 wt.% in the SBA-15. This value is higher compared to relevant values reported recently concerning other porous media such as activated carbon, natural zeolite, and halloysite. This probably occurs due to the clear mesoporous structure of the material which exhibits pore diameters in the range 5–15 nm. Mechanical tests indicated that an LDPE/xTEO@SBA-15 film exhibits improved strength properties compared to the relevant of the pure LDPE. More specifically, according to Table 1 values, the composition of wt.10% i.e., LDPE/10TEO@SBA-15, shows the best mechanical behavior. Diffusion coefficient values presented in Table 2 show higher water-vapor barrier values for LDPE/xSBA-15 and LDPE/xTEO@SBA-15 films compared with the pure LDPE films. Nevertheless, the addition of TEO in SBA-15 reduces such barriers. The material with the higher barrier was the LDPE/15TEO@SBA-15. On the other hand, according to oxygen permeability coefficient values (Pe_O2_), oxygen barrier values for LDPE/xSBA-15 and LDPE/xTEO@SBA-15 films are higher compared with the relevance of pure LDPE films. In this case, the addition of TEO in SBA-15 increases such barrier. The material with the higher barrier was the LDPE/10TEO@SBA-15. Control release measurements were carried out using the tool of the TEO diffusion coefficient (D_TEO_). Results indicated that the increase of the TEO loading led to a decrease of the diffusion kinetic constant and thus to a more controlled TEO release. Figure 7 shows a total antioxidant activity of 60–70% but no one of the tested materials could be proposed as the optimum. The diffusion rate of TEO affects the obtained antioxidant activities of active films as it is depicted by calculated EC50 values. The LDPE/10TEO@SBA-15 film with medium TEO content loaded exhibited the lower EC50 value which means the higher antioxidant activity. Finally, despite the fact that pH measurements did not show any differences between tested materials, the TBARS, Heme iron, TVC, and Sensory tests on fresh pork meat indicated clearly the LDPE/10TEO@SBA-15 material as the optimum film which could be the final product of a scaled-up process for active packaging film production.

## Figures and Tables

**Table 1:** Elastic Modulus (E), ultimate strength (σ_uts_), and elongation at break (%ε) values for all obtained LDPE/xSBA-15 and LDPE/xTEO@SBA-15 films as well as for pure LDPE film.

SAMPLE	Ε(ΜPa)	σ_uts_	%ε
LDPE	183 ± 48.3 ^a^	12.6 ± 0.5	29.3 ± 6.5
LDPE/5SBA-15	386 ± 142.2 ^b,c^	9.5 ± 3.6	14.1 ± 2.5
LDPE/10SBA-15	286 ± 31.1	9.6 ± 3.3	16.5 ± 5.7
LDPE/15SBA-15	409 ± 89.6 ^d^	10.8 ± 4.1	17.3 ± 3.8 ^e^
LDPE/5TEO@SBA-15	344 ± 12.7	11.7 ± 2.2	31.3 ± 12.9
LDPE/10TEO@SBA-15	311 ± 38.5	12.5 ± 2.1	31.9 ± 5.2
LDPE/15TEO@SBA-15	304 ± 65.4	11.2 ± 1.4	28.5 ± 11.1

^a–e^ Indexes for statistically equal mean values according to ANOVA comparison method and Tukey criteria for equal variances assumption. Significant level *p* < 0.05.

**Table 2:** WVTR, D_wv_, O.T.R. and Pe_O2_ values for all LDPE/xSBA-15 and LDPE/xTEO@SBA-15 films as well as for pure LDPE films.

	Film Thickness (mm)	WVTR (10^−7^ g·cm^−2^·s^1^)	D_wv_ (10^−4^ cm^2^/s)	Film Thickness (mm)	OTR (mL·m^−2^·Day^−1^)	P_O2_ (10^−8^ cm^2^/s)
LDPE	0.250 ± 0.012	5.85 ± 0.13	3.69 ± 0.17 ^a^	0.056 ± 0.010	3217 ± 120	2.17 ± 0.13
LDPE/5SBA-15	0.205 ± 0.011	6.91 ± 0.56	2.76 ± 0.07 ^a^	0.061 ± 0.004	2938 ± 172	1.86 ± 0.11
LDPE/10SBA-15	0.296 ± 0.014	3.32 ± 0.20	2.26 ± 0.02	0.075 ± 0.010	1641 ± 185	1.36 ± 0.54
LDPE/15SBA-15	0.270 ± 0.010	3.44 ± 0.73	1.72 ± 0.31 ^b^	0.085 ± 0.015	1688 ± 134	1.62 ± 0.37 ^c^
LDPE/5TEO@SBA-15	0.206 ± 0.012	2.33 ± 0.53	1.24 ± 0.23	0.081 ± 0.015	2938 ± 267	2.80 ± 0.15 ^c^
LDPE/10TEO@SBA-15	0.166 ± 0.016	3.74 ± 0.13	2.02 ± 0.21 ^b^	0.070 ± 0.005	2266 ± 187	1.75 ± 0.53
LDPE/15TEO@SBA-15	0.221 ± 0.013	5.35 ± 0.23	2.35 ± 0.04	0.071 ± 0.010	4003 ± 168	3.15 ± 0.13

^a–c^ Indexes for statistically equal mean values according to ANOVA comparison method and Tukey criteria for equal variances assumption. Significant level *p* < 0.05.

**Table 3:** Calculated values of wt.% TEO total released amount (%m_TEO_) diffusion coefficient of TEO (D_TEO_) molecule, desorbed equilibrium amount of TEO (q_e_), and desorption rate constant (K_2_) for all obtained LDPE/xTEO@SBA-15 active films.

	%m_TEO_ (mg)	D_TEO_ × 10^−7^ (cm^2^/s)	q_e_	K_2_ (s^−1^)	EC_50_
LDPE/5TEO@SBA-15	2.09 ± 0.17	16.7 ± 3.1	0.021 ± 0.002	0.676 ± 0.225	6.56
LDPE/10TEO@SBA-15	2.71 ± 0.22	11.3 ± 5.2	0.027 ± 0.003	0.434 ± 0.168	4.57
LDPE/15@TEO@SBA-15	4.84 ± 0.30	6.3 ± 1.92	0.052 ± 0.005	0.124 ± 0.065	5.02

**Table 4:** Calculated TBARS and heme iron content values of pork fillets wrapped with pure LDPE, and LDPE/15TEO@SBA-15 films, with respect to storage time.

TBARS	Day 0	Day 2	Day 4	Day 6	Day 8
LDPE	0.369 ^a^ ± 0.003	0.448 ^b^ ± 0.003	0.481 ^c^ ± 0.003	0.533 ^d^ ± 0.066	0.761 ^e^ ± 0.018
LDPE/10TΕO@SBA-15	0.369 ^a^ ± 0.003	0.439 ^b^ ± 0.005	0.455 ^c^ ± 0.023	0.500 ^d^ ± 0.010	0.626 ^f^ ± 0.010
Heme iron	Day 0	Day 2	Day 4	Day 6	Day 8
LDPE	11.88 ^a^ ±0.08	10.65 ^b^ ±0.04	9.33 ^d^ ±0.13	8.82 ^f^ ±0.08	7.92 ^g^ ±0.17
LDPE/10TEO@SBA-15	11.88 ^a^ ±0.08	10.92 ^c^ ±0.08	9.99 ^e^ ±0.04	9.51 ^f^ ±0.04	8.85 ^h^ ±0.13

^a–h^: Statistical significance determined by non-parametric test: *p* < 0.05 (see Table S1 for TBARS and Table S2 for heme iron). Means not sharing a common letter differ significantly at the *p* < 0.05 level, as determined by the non-parametric test.

**Table 5:** pH Evolution in minced pork meat samples wrapped with LDPE and LDPE/10TEO@SBA-15 films over an 8-day period.

pH	Day 0	Day 2	Day 4	Day 6	Day 8
LDPE	6.43 ± 0.01 ^a^	6.26 ± 0.01 ^b^	6.21 ± 0.00 ^c^	6.12 ± 0.00 ^e^	5.88 ± 0.01 ^g^
LDPE/10TEO@SBA-15	6.43 ± 0.01 ^a^	6.22 ± 0.01 ^c^	6.19 ± 0.01 ^d^	6.11 ± 0.00 ^f^	5.93 ± 0.02 ^h^

^a–h^: Statistical significance determined by non-parametric test: *p* < 0.05 (see Table S4). Means not sharing a common letter differ significantly at the *p* < 0.05 level, as determined by the non-parametric test.

**Table 6:** Calculated TVC values of minced pork meat wrapped with pure LDPE, and LDPE/10TEO@SBA-15 films and preserved for 8 days at 4 °C.

DAYS					
Sample Name	0	2	4	6	8
	logCFU/g (Avg ± SD)				
LDPE	4.03 ± 0.04 ^a^	4.53 ± 0.01 ^b^	5.07 ± 0.30 ^c^	7.36 ± 0.01 ^d^	-
LDPE/10TEO@SBA-15	4.03 ± 0.04 ^a^	4.48 ± 0.04 ^b^	4.97 ± 0.04 ^c^	7.11 ± 0.01 ^e^	-

^a–e^: Statistical significance determined by non-parametric test: *p* < 0.05 (see Table S5). Means not sharing a common letter differ significantly at the *p* < 0.05 level, as determined by the non-parametric test.

**Table 7:** Color, odor, and cohesion of wrapped minced pork meat during 6 days of storage at 4 ± 1 °C.

Color				
Sample Name	0 Day	2nd Day	4th Day	6th Day
LDPE	5.00 ± 0.00 ^a^	4.13 ± 0.52 ^b^	3.75 ± 0.53 ^c^	3.03 ± 0.47 ^d^
LDPE/10TEO@SBA-15	5.00 ± 0.00 ^a^	4.56 ± 0.40 ^b^	4.03 ± 0.61 ^c^	3.70 ± 0.55 ^e^
odor				
LDPE	5.00 ± 0.00 ^a^	4.23 ± 0.61 ^b^	3.48 ± 0.40 ^c^	2.25 ± 0.65 ^d^
LDPE/10TEO@SBA-15	5.00 ± 0.00 ^a^	4.48 ± 1.02 ^b^	4.14 ± 0.98 ^c^	3.89 ± 0.98 ^e^
cohesion				
LDPE	5.00 ± 0.00 ^a^	4.09 ± 0.55 ^b^	3.10 ± 0.39 ^d^	2.28 ± 0.63 ^f^
LDPE/10TEO@SBA-15	5.00 ± 0.00 ^a^	4.71 ± 0.36 ^c^	4.10 ± 0.75 ^e^	3.58 ± 0.68 ^g^

^a–g^: Statistical significance determined by non-parametric test: *p* < 0.05 (see Table S6). Means not sharing a common letter differ significantly at the *p* < 0.05 level, as determined by the non-parametric test.

## Data Availability

The datasets generated for this study are available on request to the corresponding author.

## Supplementary Materials

The following supporting information can be downloaded at: https://www.mdpi.com/article/10.3390/nano14050423/s1, Table S1: Statistical analysis on lipid oxidation; Table S2: Statistical analysis on Heme Fe; Table S3: Correlation Pearson Heme Fe and Lipid Oxidation; Table S4: Statistical Analysis on pH; Table S5: Statistical Analysis on TVC tests; Table S6: Statistical Analysis of Sensory Evaluation Data.

## References

[1] Hamam M., Chinnici G., Di Vita G., Pappalardo G., Pecorino B., Maesano G., D’Amico M. (2021). Circular Economy Models in Agro-Food Systems: A Review. Sustainability.

[2] Scarano P., Sciarrillo R., Tartaglia M., Zuzolo D., Guarino C. (2022). Circular Economy and Secondary Raw Materials from Fruits as Sustainable Source for Recovery and Reuse. A Review. Trends Food Sci. Technol..

[3] Asgher M., Qamar S.A., Bilal M., Iqbal H.M.N. (2020). Bio-Based Active Food Packaging Materials: Sustainable Alternative to Conventional Petrochemical-Based Packaging Materials. Food Res. Int..

[4] Fernández-Acero F.J., Amil-Ruiz F., Durán-Peña M.J., Carrasco R., Fajardo C., Guarnizo P., Fuentes-Almagro C., Vallejo R.A. (2019). Valorisation of the Microalgae Nannochloropsis Gaditana Biomass by Proteomic Approach in the Context of Circular Economy. J. Proteomics.

[5] Viaggi D. (2022). Agricultural Waste Management and Valorisation in the Context of the Circular Bioeconomy: Exploring the Potential of Biomass Value Webs. Curr. Opin. Environ. Sci. Health.

[6] Ahmed M.W., Haque M.A., Mohibbullah M., Khan M.S.I., Islam M.A., Mondal M.H.T., Ahmmed R. (2022). A Review on Active Packaging for Quality and Safety of Foods: Current Trends, Applications, Prospects and Challenges. Food Packag. Shelf Life.

[7] Barman M., Mahmood S., Augustine R., Hasan A., Thomas S., Ghosal K. (2020). Natural Halloysite Nanotubes/Chitosan Based Bio-Nanocomposite for Delivering Norfloxacin, an Anti-Microbial Agent in Sustained Release Manner. Int. J. Biol. Macromol..

[8] Plant Extracts-Based Food Packaging Films—Natural Materials for Food Packaging Application—Wiley Online Library. https://onlinelibrary.wiley.com/doi/10.1002/9783527837304.ch2.

[9] Carpena M., Nuñez-Estevez B., Soria-Lopez A., Garcia-Oliveira P., Prieto M.A. (2021). Essential Oils and Their Application on Active Packaging Systems: A Review. Resources.

[10] Ribeiro-Santos R., Andrade M., de Melo N.R., Sanches-Silva A. (2017). Use of Essential Oils in Active Food Packaging: Recent Advances and Future Trends. Trends Food Sci. Technol..

[11] Sharma S., Barkauskaite S., Jaiswal A.K., Jaiswal S. (2021). Essential Oils as Additives in Active Food Packaging. Food Chem..

[12] Nieto G. (2020). A Review on Applications and Uses of Thymus in the Food Industry. Plants.

[13] Kowalczyk A., Przychodna M., Sopata S., Bodalska A., Fecka I. (2020). Thymol and Thyme Essential Oil—New Insights into Selected Therapeutic Applications. Molecules.

[14] Varghese S.A., Siengchin S., Parameswaranpillai J. (2020). Essential Oils as Antimicrobial Agents in Biopolymer-Based Food Packaging—A Comprehensive Review. Food Biosci..

[15] Zubair M., Shahzad S., Hussain A., Pradhan R.A., Arshad M., Ullah A. (2022). Current Trends in the Utilization of Essential Oils for Polysaccharide- and Protein-Derived Food Packaging Materials. Polymers.

[16] Chacha J.S., Ofoedu C.E., Xiao K. (2022). Essential Oil-Based Active Polymer-Based Packaging System: A Review of Its Effect on the Antimicrobial, Antioxidant, and Sensory Properties of Beef and Chicken Meat. J. Food Process. Preserv..

[17] Plastic Films in Food Packaging—1st Edition. https://shop.elsevier.com/books/plastic-films-in-food-packaging/ebnesajjad/978-1-4557-3112-1.

[18] Saucedo-Zuñiga J.N., Sánchez-Valdes S., Ramírez-Vargas E., Guillen L., Ramos-deValle L.F., Graciano-Verdugo A., Uribe-Calderón J.A., Valera-Zaragoza M., Lozano-Ramírez T., Rodríguez-González J.A. (2021). Controlled Release of Essential Oils Using Laminar Nanoclay and Porous Halloysite/Essential Oil Composites in a Multilayer Film Reservoir. Microporous Mesoporous Mater..

[19] de Oliveira L.H., Trigueiro P., Souza J.S.N., de Carvalho M.S., Osajima J.A., da Silva-Filho E.C., Fonseca M.G. (2022). Montmorillonite with Essential Oils as Antimicrobial Agents, Packaging, Repellents, and Insecticides: An Overview. Colloids Surf. B Biointerfaces.

[20] Giannakas A., Tsagkalias I., Achilias D.S., Ladavos A. (2017). A Novel Method for the Preparation of Inorganic and Organo-Modified Montmorillonite Essential Oil Hybrids. Appl. Clay Sci..

[21] Salmas C.E., Giannakas A.E., Karabagias V.K., Moschovas D., Karabagias I.K., Gioti C., Georgopoulos S., Leontiou A., Kehayias G., Avgeropoulos A. (2023). Development and Evaluation of a Novel-Thymol@Natural-Zeolite/Low-Density-Polyethylene Active Packaging Film: Applications for Pork Fillets Preservation. Antioxidants.

[22] Giannakas A. (2020). Na-Montmorillonite vs. Organically Modified Montmorillonite as Essential Oil Nanocarriers for Melt-Extruded Low-Density Poly-Ethylene Nanocomposite Active Packaging Films with a Controllable and Long-Life Antioxidant Activity. Nanomaterials.

[23] Giannakas A.E., Salmas C.E., Moschovas D., Karabagias V.K., Karabagias I.K., Baikousi M., Georgopoulos S., Leontiou A., Katerinopoulou K., Zafeiropoulos N.E. (2023). Development, Characterization, and Evaluation as Food Active Packaging of Low-Density-Polyethylene-Based Films Incorporated with Rich in Thymol Halloysite Nanohybrid for Fresh “Scaloppini” Type Pork Meat Fillets Preservation. Polymers.

[24] Giannakas A.E., Karabagias V.K., Moschovas D., Leontiou A., Karabagias I.K., Georgopoulos S., Karydis-Messinis A., Zaharioudakis K., Andritsos N., Kehayias G. (2023). Thymol@activated Carbon Nanohybrid for Low-Density Polyethylene-Based Active Packaging Films for Pork Fillets’ Shelf-Life Extension. Foods.

[25] Sullivan D.J., O’Mahony T.F., Cruz-Romero M.C., Cummins E., Kerry J.P., Morris M.A. (2021). The Use of Porous Silica Particles as Carriers for a Smart Delivery of Antimicrobial Essential Oils in Food Applications. ACS Omega.

[26] Gámez E., Elizondo-Castillo H., Tascon J., García-Salinas S., Navascues N., Mendoza G., Arruebo M., Irusta S. (2020). Antibacterial Effect of Thymol Loaded SBA-15 Nanorods Incorporated in PCL Electrospun Fibers. Nanomaterials.

[27] Himed L., Merniz S., Monteagudo-Olivan R., Barkat M., Coronas J. (2019). Antioxidant Activity of the Essential Oil of *Citrus Limon* before and after Its Encapsulation in Amorphous SiO_2_. Sci. Afr..

[28] Popovici R.F., Seftel E.M., Mihai G.D., Popovici E., Voicu V.A. (2011). Controlled Drug Delivery System Based on Ordered Mesoporous Silica Matrices of Captopril as Angiotensin-Converting Enzyme Inhibitor Drug. J. Pharm. Sci..

[29] Ukmar T., Planinšek O. (2010). Ordered Mesoporous Silicates as Matrices for Controlled Release of Drugs. Acta Pharm..

[30] Gargiulo N., Attianese I., Buonocore G.G., Caputo D., Lavorgna M., Mensitieri G., Lavorgna M. (2013). α-Tocopherol Release from Active Polymer Films Loaded with Functionalized SBA-15 Mesoporous Silica. Microporous Mesoporous Mater..

[31] Larki A., Saghanezhad S.J., Ghomi M. (2021). Recent Advances of Functionalized SBA-15 in the Separation/Preconcentration of Various Analytes: A Review. Microchem. J..

[32] Nandiyanto A.B.D., Kim S.-G., Iskandar F., Okuyama K. (2009). Synthesis of Spherical Mesoporous Silica Nanoparticles with Nanometer-Size Controllable Pores and Outer Diameters. Microporous Mesoporous Mater..

[33] Materials|Free Full-Text|SBA-15 Mesoporous Silica as Catalytic Support for Hydrodesulfurization Catalysts—Review. https://www.mdpi.com/1996-1944/6/9/4139.

[34] Mavrogiorgou A., Baikousi M., Costas V., Mouzourakis E., Deligiannakis Y., Karakassides M.A., Louloudi M. (2016). Mn-Schiff Base Modified MCM-41, SBA-15 and CMK-3 NMs as Single-Site Heterogeneous Catalysts: Alkene Epoxidation with H2O2 Incorporation. J. Mol. Catal. A Chem..

[35] Giannakas A., Giannakas A., Ladavos A. (2012). Preparation and Characterization of Polystyrene/Organolaponite Nanocomposites. Polym. Plast. Technol. Eng..

[36] Giannakas A.E., Salmas C.E., Leontiou A., Baikousi M., Moschovas D., Asimakopoulos G., Zafeiropoulos N.E., Avgeropoulos A. (2021). Synthesis of a Novel Chitosan/Basil Oil Blend and Development of Novel Low Density Poly Ethylene/Chitosan/Basil Oil Active Packaging Films Following a Melt-Extrusion Process for Enhancing Chicken Breast Fillets Shelf-Life. Molecules.

[37] Giannakas A.E., Salmas C.E., Moschovas D., Baikousi M., Kollia E., Tsigkou V., Karakassides A., Leontiou A., Kehayias G., Avgeropoulos A. (2022). Nanocomposite Film Development Based on Chitosan/Polyvinyl Alcohol Using ZnO@Montmorillonite and ZnO@Halloysite Hybrid Nanostructures for Active Food Packaging Applications. Nanomaterials.

[38] Helmroth E., Rijk R., Dekker M., Jongen W. (2002). Predictive Modelling of Migration from Packaging Materials into Food Products for Regulatory Purposes. Trends Food Sci. Technol..

[39] Mishra K., Ojha H., Chaudhury N.K. (2012). Estimation of Antiradical Properties of Antioxidants Using DPPH Assay: A Critical Review and Results. Food Chem..

[40] EC50 Estimation of Antioxidant Activity in DPPH Assay Using Several Statistical Programs—ScienceDirect. https://www.sciencedirect.com/science/article/pii/S0308814612016020.

[41] Beretta G., Granata P., Ferrero M., Orioli M., Maffei Facino R. (2005). Standardization of Antioxidant Properties of Honey by a Combination of Spectrophotometric/Fluorimetric Assays and Chemometrics. Anal. Chim. Acta.

[42] Brand-Williams W., Cuvelier M.E., Berset C. (1995). Use of a Free Radical Method to Evaluate Antioxidant Activity. LWT Food Sci. Technol..

[43] Tarladgis B.G., Watts B.M., Younathan M.T., Dugan L. (1960). A Distillation Method for the Quantitative Determination of Malonaldehyde in Rancid Foods. J. Am. Oil. Chem. Soc..

[44] Clark E.M., Mahoney A.W., Carpenter C.E. (1997). Heme and Total Iron in Ready-to-Eat Chicken. J. Agric. Food Chem..

[45] Janisch S., Krischek C., Wicke M. (2011). Color Values and Other Meat Quality Characteristics of Breast Muscles Collected from 3 Broiler Genetic Lines Slaughtered at 2 Ages. Poult. Sci..

[46] Assanti E., Karabagias V.K., Karabagias I.K., Badeka A., Kontominas M.G. (2021). Shelf Life Evaluation of Fresh Chicken Burgers Based on the Combination of Chitosan Dip and Vacuum Packaging under Refrigerated Storage. J. Food Sci. Technol..

[47] Hematizad I., Khanjari A., Basti A.A., Karabagias I.K., Noori N., Ghadami F., Gholami F., Teimourifard R. (2021). In Vitro Antibacterial Activity of Gelatin-Nanochitosan Films Incorporated with Zataria Multiflora Boiss Essential Oil and Its Influence on Microbial, Chemical, and Sensorial Properties of Chicken Breast Meat during Refrigerated Storage. Food Packag. Shelf Life.

[48] Achilias D.S., Gerakis K., Giliopoulos D.J., Triantafyllidis K.S., Bikiaris D.N. (2016). Effect of High Surface Area Mesoporous Silica Fillers (MCF and SBA-15) on Solid State Polymerization of PET. Eur. Polym. J..

[49] Albayati T.M., Salih I.K., Alazzawi H.F. (2019). Synthesis and Characterization of a Modified Surface of SBA-15 Mesoporous Silica for a Chloramphenicol Drug Delivery System. Heliyon.

[50] Giannakas A., Salmas C., Leontiou A., Tsimogiannis D., Oreopoulou A., Braouhli J. (2019). Novel LDPE/Chitosan Rosemary and Melissa Extract Nanostructured Active Packaging Films. Nanomaterials.

[51] Martínez-Camacho A.P., Cortez-Rocha M.O., Graciano-Verdugo A.Z., Rodríguez-Félix F., Castillo-Ortega M.M., Burgos-Hernández A., Ezquerra-Brauer J.M., Plascencia-Jatomea M. (2013). Extruded Films of Blended Chitosan, Low Density Polyethylene and Ethylene Acrylic Acid. Carbohydr. Polym..

[52] Díez-Rodríguez T.M., Blázquez-Blázquez E., Antunes N.L.C., Ribeiro M.R., Pérez E., Cerrada M.L. (2022). Nanocomposites of PCL and SBA-15 Particles Prepared by Extrusion: Structural Characteristics, Confinement of PCL Chains within SBA-15 Nanometric Channels and Mechanical Behavior. Polymers.

[53] Maranhão S.L.A., Cides da Silva L.C., Michels A.F., Horowitz F., Matos J.R., Fantini M.C.A. (2014). Structure and Morphology of SBA-15 Thin Films on Different Substrates. Braz. J. Phys..

[54] Chen Z., Song C., Bai R., Wei Z., Zhang F. (2012). Effects of Mesoporous SBA-15 Contents on the Properties of Polystyrene Composites via in-Situ Emulsion Polymerization. J. Polym. Res..

[55] Celina M.C., Quintana A. (2018). Oxygen Diffusivity and Permeation through Polymers at Elevated Temperature. Polymer.

[56] Participation E. Commission Regulation (EC) No 2073/2005 of 15 November 2005 on Microbiological Criteria for Foodstuffs (Text with EEA Relevance). https://www.legislation.gov.uk/eur/2005/2073/contents.

[57] Huang L., Zhao J., Chen Q., Zhang Y. (2013). Rapid Detection of Total Viable Count (TVC) in Pork Meat by Hyperspectral Imaging. Food Res. Int..

[58] Ruiz-Capillas C., Herrero A.M., Pintado T., Delgado-Pando G. (2021). Sensory Analysis and Consumer Research in New Meat Products Development. Foods.

[59] Estimation of Sensory Pork Loin Tenderness Using Warner-Bratzler Shear Force and Texture Profile Analysis Measurements. https://www.animbiosci.org/journal/view.php?doi=10.5713/ajas.15.0482.

[60] Tomasevic I., Djekic I., Font-i-Furnols M., Terjung N., Lorenzo J.M. (2021). Recent Advances in Meat Color Research. Curr. Opin. Food Sci..

[61] Bassey A.P., Chen Y., Boateng E.F., Zhang Y., Diao X., Nasiru M.M., Tang C., Ye K., Li C., Zhou G. (2022). Evaluation of Physicochemical, Microbiological, and Sensory Profiles of Vacuum-Packed Cooked Low-Salt Pork Belly under Refrigeration and Room-Temperature Storage. LWT.

