# Supplementary material for: Low-Density Polyethylene-Based Novel Active Packaging Film for Food Shelf-Life Extension via Thyme-Oil Control Release from SBA-15 Nanocarrier
Source: Nanomaterials (Basel). 2024 Feb 26;14(5):423. doi: 10.3390/nano14050423 (PMC10933968; doi:10.3390/nano14050423)
Supplement: Supplementary file 1 [file nanomaterials-14-00423-s001.zip › nanomaterials-2861607-supplementary.pdf]

Table S1. Statistical analysis on lipid oxidation.

Hypothesis Test Summary

	Null Hypothesis	Test	Sig.[a,b]	Decision
1	The distribution of Day_0 is the same across categories of Treatment.	Independent-Samples Kruskal-Wallis Test	1.000	Retain the null hypothesis.
2	The distribution of Day_2 is the same across categories of Treatment.	Independent-Samples Kruskal-Wallis Test	0.513	Retain the null hypothesis.
3	The distribution of Day_4 is the same across categories of Treatment.	Independent-Samples Kruskal-Wallis Test	0.127	Retain the null hypothesis.
4	The distribution of Day_6 is the same across categories of Treatment.	Independent-Samples Kruskal-Wallis Test	0.513	Retain the null hypothesis.
5	The distribution of Day_8 is the same across categories of Treatment.	Independent-Samples Kruskal-Wallis Test	0.050	Reject the null hypothesis.

a. The significance level is ,050.
b. Asymptotic significance is displayed.

Table S2. Statistical analysis on Heme Fe.

Hypothesis Test Summary

	Null Hypothesis	Test	Sig.[a,b]	Decision
1	The distribution of Day_0 is the same across categories of Treatment.	Independent-Samples Kruskal-Wallis Test	1,000	Retain the null hypothesis.
2	The distribution of Day_2 is the same across categories of Treatment.	Independent-Samples Kruskal-Wallis Test	,050	Reject the null hypothesis.
3	The distribution of Day_4 is the same across categories of Treatment.	Independent-Samples Kruskal-Wallis Test	,050	Reject the null hypothesis.
4	The distribution of Day_6 is the same across categories of Treatment.	Independent-Samples Kruskal-Wallis Test	,513	Retain the null hypothesis.
5	The distribution of Day_8 is the same across categories of Treatment.	Independent-Samples Kruskal-Wallis Test	,050	Reject the null hypothesis.

a. The significance level is ,050.

b. Asymptotic significance is displayed.

Table S3. Correlation Pearson Heme Fe and Lipid Oxidation.

Correlations

		Heme_Day_8	Lipid_Day_8
Heme_Day_8	Pearson Correlation	1	-,930**
	Sig. (2-tailed)		,007
	Sum of Squares and Cross-products	1,377	-,183
	Covariance	,275	-,037
	N	6	6
Lipid_Day_8	Pearson Correlation	-,930**	1
	Sig. (2-tailed)	,007	
	Sum of Squares and Cross-products	-,183	,028
	Covariance	-,037	,006
	N	6	6

**. Correlation is significant at the 0.01 level (2-tailed).

Table S4. Statistical Analysis on pH.

Hypothesis Test Summary

	Null Hypothesis	Test	Sig.[a,b]	Decision
1	The distribution of Day_0 is the same across categories of Treatment.	Independent-Samples Kruskal-Wallis Test	1,000	Retain the null hypothesis.
2	The distribution of Day_2 is the same across categories of Treatment.	Independent-Samples Kruskal-Wallis Test	,034	Reject the null hypothesis.
3	The distribution of Day_4 is the same across categories of Treatment.	Independent-Samples Kruskal-Wallis Test	,025	Reject the null hypothesis.
4	The distribution of Day_6 is the same across categories of Treatment.	Independent-Samples Kruskal-Wallis Test	,25	Retain the null hypothesis.
5	The distribution of Day_8 is the same across categories of Treatment.	Independent-Samples Kruskal-Wallis Test	,050	Reject the null hypothesis.

a. The significance level is ,050.

b. Asymptotic significance is displayed.

Table S5. Statistical Analysis on TVC tests.

Hypothesis Test Summary

	Null Hypothesis	Test	Sig.[a,b]	Decision

1	The distribution of Day_0 is the same across categories of Treatment.	Independent-Samples Kruskal-Wallis Test	1,000	Retain the null hypothesis.
2	The distribution of Day_2 is the same across categories of Treatment.	Independent-Samples Kruskal-Wallis Test	,513	Retain the null hypothesis.
3	The distribution of Day_4 is the same across categories of Treatment.	Independent-Samples Kruskal-Wallis Test	,513	Retain the null hypothesis.
4	The distribution of Day_6 is the same across categories of Treatment.	Independent-Samples Kruskal-Wallis Test	,025	Reject the null hypothesis.

a. The significance level is ,050.

b. Asymptotic significance is displayed.

Table S6. Statistical Analysis of Sensory Evaluation Data.

Odor

Hypothesis Test Summary

	Null Hypothesis	Test	Sig.[a,b]	Decision
1	The distribution of Odor_Day_0 is the same across categories of Treatment_Odor_Day.	Independent-Samples Kruskal-Wallis Test	1,000	Retain the null hypothesis.
2	The distribution of Odor_Day_2 is the same across categories of Treatment_Odor_Day.	Independent-Samples Kruskal-Wallis Test	,110	Retain the null hypothesis.
3	The distribution of Odor_Day_4 is the same across categories of Treatment_Odor_Day.	Independent-Samples Kruskal-Wallis Test	,365	Retain the null hypothesis.
4	The distribution of Odor_Day_6 is the same across categories of Treatment_Odor_Day.	Independent-Samples Kruskal-Wallis Test	,014	Reject the null hypothesis.

a. The significance level is ,050.

b. Asymptotic significance is displayed.

Colour

Hypothesis Test Summary

	Null Hypothesis	Test	Sig.[a,b]	Decision
1	The distribution of Colour_Day0 is the same across categories of Treatment_Colour_Day.	Independent-Samples Kruskal-Wallis Test	1,000	Retain the null hypothesis.

2	The distribution of Colour_Day2 is the same across categories of Treatment_Colour_Day.	Independent-Samples Kruskal-Wallis Test	,110	Retain the null hypothesis.
3	The distribution of Colour_Day4 is the same across categories of Treatment_Colour_Day.	Independent-Samples Kruskal-Wallis Test	,365	Retain the null hypothesis.
4	The distribution of Colour_Day6 is the same across categories of Treatment_Colour_Day.	Independent-Samples Kruskal-Wallis Test	,014	Reject the null hypothesis.

a. The significance level is ,050.

b. Asymptotic significance is displayed.

Hypothesis Test Summary

	Null Hypothesis	Test	Sig.[a,b]	Decision
1	The distribution of Cohetion_Day0 is the same across categories of Treatment_Cohetion_Day.	Independent-Samples Kruskal-Wallis Test	1,000	Retain the null hypothesis.
2	The distribution of Cohetion_Day2 is the same across categories of Treatment_Cohetion_Day.	Independent-Samples Kruskal-Wallis Test	,010	Reject the null hypothesis.
3	The distribution of Cohetion_Day4 is the same across categories of Treatment_Cohetion_Day.	Independent-Samples Kruskal-Wallis Test	,008	Reject the null hypothesis.
4	The distribution of Cohetion_Day6 is the same across categories of Treatment_Cohetion_Day.	Independent-Samples Kruskal-Wallis Test	,002	Reject the null hypothesis.

a. The significance level is ,050.

b. Asymptotic significance is displayed.
